# The Roles of Macrophages and Nitric Oxide in Interleukin-3-Enhanced HSV-Sr39tk-Mediated Prodrug Therapy

**DOI:** 10.1371/journal.pone.0056508

**Published:** 2013-02-18

**Authors:** Ching-Fang Yu, Ji-Hong Hong, Chi-Shiun Chiang

**Affiliations:** 1 Department of Biomedical Engineering and Environmental Sciences, National Tsing-Hua University, Hsinchu, Taiwan; 2 Department of Radiation Oncology, Chang-Gung Memorial Hospital, Taoyuan, Taiwan; 3 Department of Medical Imaging and Radiological Science, Chang Gung University, Taiwan; University of Pécs Medical School, Hungary

## Abstract

The herpes simplex virus thymidine kinase/ganciclovir (HSV-sr39tk/GCV) system is a well-established prodrug system used in cancer gene therapy. However, this system is currently not effective enough to eradicate malignant tumors completely. This study aimed to evaluate whether co-expression of interleukin-3 (IL-3) could enhance the anti-tumor activity of HSV-sr39tk/GCV prodrug gene therapy using a murine TRAMP-C1 prostate tumor model. *In vitro* results demonstrated that HSV-sr39tk-transfected cells exhibited enhanced sensitivity to the GCV prodrug, which was not affected by co-expression of the mIL-3 gene. However, *in vivo* studies showed that co-expression of the mIL-3 gene significantly increased the HSV-sr39tk/GCV-induced tumor growth delay and even cured the tumor. The TRAMP-C1-specific immune response of spleen lymphocytes from mice bearing HSV-sr39tk- and IL-3-expressing TRAMP-C1 tumors was measured by ELISA. Results showed that IL-3-activated IL-4-dominant lymphocytes became IFN-γ- dominant lymphocytes after combined HSV-sr39tk/GCV therapy. The efficacy of combined therapies on tumor regression was reduced when macrophages populations were depleted by carrageenan or NO production was inhibited by administration of the iNOS inhibitor, L-NAME. These results suggest that utilizing a bicistronic vector to express HSV-sr39tk and the IL-3 gene induced an enhanced macrophage- or NO-dependent anti-tumor effect.

## Introduction

Proposed by Moolten in 1986, the activation of a suicide gene encoding an enzyme protein that is nontoxic to genetically modified cells has become an extensively adopted approach for prodrug gene therapies designed to treat cancer [1], [2]. Herpes simplex virus type 1 thymidine kinase (HSV-tk)/ganciclovir (GCV) is the most widely used suicide gene/prodrug system in preclinical and clinical studies. HSV-tk has broad substrate specificity and is able to monophosphorylate the prodrug, the thymidine analogue, around 1000 times more efficiently than mammalian thymidine kinase [3]. In addition to killing genetically modified cells, the bystander effect also plays an important role in this system, in that non-modified cells are killed indirectly via gap junctions between cells and/or by the immune-mediated anti-tumor effects of macrophages, T lymphocytes or natural killer (NK) cells [4], [5], [6], [7].

Although the bystander effect promotes tumor cell death, inefficient activation of GCV by HSV-tk and prodrug-associated negative side effects limit the clinical efficacy of this system. Using HSV-tk mutants is one approach to improving the activity and specificity for GCV [8], [9]. Two mutants, dm30-tk and sr39-tk, have been extensively studied in both *in vitro* and *in vivo* models. These studies have demonstrated that cells transfected with either of the mutant enzymes were more sensitive to the cytotoxic effects of GCV and acyclovir (ACV) when compared to cells transfected with wild-type HSV-tk. In addition to improving GCV activation, combining the HSV-tk/GCV system with other strategies, such as cytokine therapy, has been demonstrated in several tumor systems to be more effective than using a single treatment [10], [11], [12], [13].

Interleukin-3 (IL-3) is well characterized as a multicolony-stimulating factor that affects the growth of most hemopoietic lineages and is produced by cytotoxic and helper T lymphocytes in mice [14], [15]. IL-3 can enhance antigen presentation by dendritic cells and activate macrophages to increase the expression of class II MHC molecules and interleukin-1 (IL-1) [16]. Tumor secreting IL-3 recruits more macrophages, polymorphonuclear leukocytes, and T cells into the local microenvironment [17], [18]. In a mouse lung carcinoma model, IL-3 enhanced tumor rejection by enhancing cytotoxic effectors through a mechanism that required CD4^+^ cells [19]. Our previous studies have demonstrated that expression of IL-3 within tumors can improve host immunity thereby enhancing eradication of tumor cells, even of classically non-immunogenic tumors, and enhance the effects of radiation therapy to develop a long-term state of immunity after regression of the primary tumor [20].

In the current study, we investigated whether intratumoral expression of murine IL-3 (mIL-3) enhances the anti-tumor effect of the HSV-tk/GCV system in a murine TRAMP-C1 prostate cancer model *in vivo*. The results demonstrated that bicistronic expression of HSV-sr39tk and the mIL-3 gene followed by GCV administration induced a more effective anti-tumor response compared to that achieved with either HSV-sr39tk/GCV or mIL-3 therapy alone. In addition, we reveal that the anti-tumor mechanism of the combined HSV-sr39tk/GCV and mIL-3 therapy was associated with macrophages or nitric oxide (NO)-dependent T helper 1 (Th1) immune response.

## Materials and Methods

### Mice

Six- to eight-week-old C57BL/6J male mice were purchased from the National Laboratory Animal Center, Taiwan. C.B17/Icr-scid mice were purchased from the Laboratory Animal Center of National Taiwan University College of Medicine, Taiwan. The recommendations of the approved guide for the care and use of laboratory animals by the Institutional Animal Care and Use Committee (IACUC) of National Tsing Hua University, Taiwan (approved number: IACUC:10012), were followed at all times. All surgery was performed under ketamine/xylazine anesthesia, and all efforts were made to minimize suffering.

### Cell Line Cultures

TRAMP-C1 mouse prostate cancer cell line was purchased from American Type Culture Collection (CRL-2730) and RAW264.7 mouse macrophage cell line was purchased from Bioresource Collection and Research Center (BCRC-60001), Taiwan. TRAMP-C1 cells were maintained in Dulbecco’s modified Eagle’s medium (DMEM; Gibco, Long Island, NY) with 10% fetal bovine serum (FBS; Gibco), 1% penicillin-streptomycin (Gibco), 5 µg/ml insulin (Sigma-Aldrich, St. Louis, MO, USA), and 10^−8^ M dehydrotestosterone (DHT; Sigma-Aldrich). RAW264.7 cells were maintained in Dulbecco’s modified Eagle’s medium with 10% fetal bovine serum, 1% penicillin-streptomycin. Cell lines were incubated at 37°C in humidified 5% CO_2_/air atmosphere.

### Plasmids Construction and Cell Transfection

The bicistronic expression vector, IRES (BD Clontech, Mountain View, CA, USA), was used to express full length of HSV-sr39tk or/and mIL-3 genes. HSV-sr39tk DNA fragments were amplified from SP72-sr39tk plasmid by PCR with specific primers (5′-primer with NehI site: 5′-ATGCTAGCATGGCTTCGTACCCCGGCCAT-3′ and 3′-primer with XhoI site: 5′-ATCTCGAGTCAGTTAGCCTCCCCCATCTC-3′). After restriction enzyme digestion, sr39tk DNA fragments were cloned into IRES or IRES-mIL3 vector to generate IRES-sr39tk or IRES-mIL3-sr39tk plasmid. The three constructed plasmids, IRES-sr39tk, IRES-mIL3 and IRES-mIL3-sr39tk, were transfected into TRAMP-C1 cell line by Effectene Transfection Reagent (Qiagen, Hilden, Germany). After 48 h of transfection, cells were selected in a culture medium containing 1.5 mg/ml G418 for two weeks. The transfected cells were called TRAMP-C1/sr39tk, TRAMP-C1/mIL3, or TRAMP-C1/mIL3-sr39tk.

### RNA Isolation and RT-PCR

Total RNA was isolated from cells by TRIzol reagent according to manufacturer’s protocol (Invitrogen, Carlsbad, CA, USA). Two µg of total RNA was reversely transcripted into cDNA by Omniscript reverse transcriptase kit (Qiagen). The primer pairs for sr39tk and mIL-3 were: (forward, 5′-CGCCGCCCTCCTGTGCTACC-3′; and reverse, 5′-GGGCCCGCGTTGCTCTG G-3′) and (forward, 5′-TGCGCTGCCAGGGGTCTT-3′; and reverse, 5′-ATTCCACG GTTCCACGGTTAGG-3′), respectively.

### GCV Sensitive Assay

TRAMP-C1 and transfected cells were seeded in 96-well culture plate (1000 cells/well) and cultured overnight. After 24 h, medium were exchanged for fresh medium containing serial diluted GCV (Sigma-Aldrich) concentration from 0 to 200 µg/mL. After incubation for three days, viability of cells were quantified by MTT (3-[4,5-dimethylthiazol-2-yl]-2,5-diphenyl tetrazolium bromide; Sigma-Aldrich) assay, and O.D. values were measured at 570 nm wavelength. The percentage of surviving cells was calculated as a percentage of absorbance found in the GCV-treated cells divided by that found in the cells without GCV treatment.

### Preparation of Peritoneal Macrophages and Assays for IFN-γ Cytokine Production in vitro

Mice were intraperitoneally (i.p.) injected with 2 mL each of 3% fluid thioglycolate medium (Sigma-Aldrich) and sacrificed four days later, followed by peritoneal lavage with 10 mL of cold PBS twice. About 90% of collected peritoneal cells were CD11b positive as determined by staining with PE-conjugated anti-Mac-I antibody. 2×10^5^ macrophages were co-cultured with 2×10^5^ tumor cell lines in 1.0 mL of culture media in 24-well tissue culture plates, and treated with GCV for 3 days at 37°C. The concentration of IFN-γ in the media was quantified using IFN-γ immunoassay kit (BD Pharmingen, San Jose, CA, USA).

### Animal Study

TRAMP-C1 (1.5×10^6^), TRAMP-C1/sr39tk (1.5×10^6^), TRAMP-C1/mIL3 (3×10^6^) and TRAMP-C1/mIL3-sr39tk (3×10^6^) tumor cells were injected subcutaneously into the right flanks of C57BL/6J mice to establish tumor model. Tumor volumes were measured every two days with a caliper until the experiment was completed. When tumor diameter reached 5 mm, each group of mice was randomly divided into two subgroups: PBS and GCV treatment. GCV (33 mg/kg) were dissolved in 100 µl PBS and administered intraperitoneally twice per day for five consecutive days.

Monocyte/macrophages lineage was depleted as described [21]. Briefly, carrageenan was dissolved in PBS at 10 mg/ml. Carrageenan (2 mg/mouse; Sigma-Aldrich) were i.p. injected at 7, 3, 1 day prior to tumor inoculation, and continued twice per week after tumor implantation until mice sacrificed.

### Immune Response Assay

Spleens were taken from tumor-bearing mice after 14 days of GCV administration and dispersed by pushing through cell strainer. Red blood cells were lysed by RBS lysis buffer (BioLegend, San Diego, CA, USA). Splenocytes were re-suspended in RPMI 1640 with 10% FBS and 1% penicillin-streptomycin and re-stimulated by adding irradiated (70 Gy) TRAMP-C1 cell at 10∶1 ratio for 48 h. Cell supernatants were harvested for IFN-γ and IL-4 immunoassay kits (BD Pharmingen).

### Flow Cytometry

The phenotype of tumor-infiltrating cells was evaluated by specific antibodies. First, tumors were digested into single cell suspension by dispase (BD Pharmingen) and filtered through 70 µm cell strainer. To avoid non-specific binding, cells were blocked by anti-mouse CD16/CD32 antibody (BD Pharmingen) and serum before antibody staining and incubated on ice for 30 min. After washed twice, cell suspensions were stained by PE-CD11b, FITC-Gr-1 (BD Pharmingen), FITC-F4/80, FITC-CD68, isotype control antibodies (Serotec) on ice for 1 h, and then washed twice. For intracellular staining, cell surface staining were performed first and then fixed by Fixation buffer (BioLegend) for 20 min on ice. Cells were washed by Permeabilization buffer (BioLegend) twice and resuspended in Permeabilization buffer for intracellular staining. FACS analysis was performed on FACSCanto II flow cytometer (Becton Dickinson) and data were analyzed by FACSDiva software.

### Immunohistochemical Analysis

Tumor tissue samples were embedded in OCT compound (Sakura Finetek, Torrance, CA, USA), snap-frozen in liquid nitrogen and stored in −80°C. Cryostat sections of frozen tissues were fixed in cold methanol for 5 min, followed by two rinses in PBS. To avoid non-specific staining, tumor tissue slides were blocked and permeabilized with blocking buffer (4% FBS and 1% normal goat serum in PBS) containing 0.01% Tween-20 and 0.1%Triton for 30 min at room temperature. The slides were subsequently incubated with rat anti-mouse CD11b, CD68, and rabbit anti-mouse iNOS (BD Pharmingen) antibodies. After 24 h, slides were washed and incubated with secondary antibodies conjugated with Alexa Fluor 488 or 594 (Invitrogen) for 1 h. After wash, slides were mounted with DAPI (Invitrogen) to visualize nuclei.

### Statistics

Where indicated, the results of tests of significance are reported as *P-*values <0.05 and are derived from the Student’s *t* test using a two-tailed distribution or one-way ANOVA. The *P*-values were calculated using the GraphPad Prism software version 5 package (GraphPad Software, Inc. San Diego, CA).

## Results

### Characteristics of mIL-3- and HSV-sr39tk-transfected Cell Lines in vitro and in vivo

**Figure 1:**
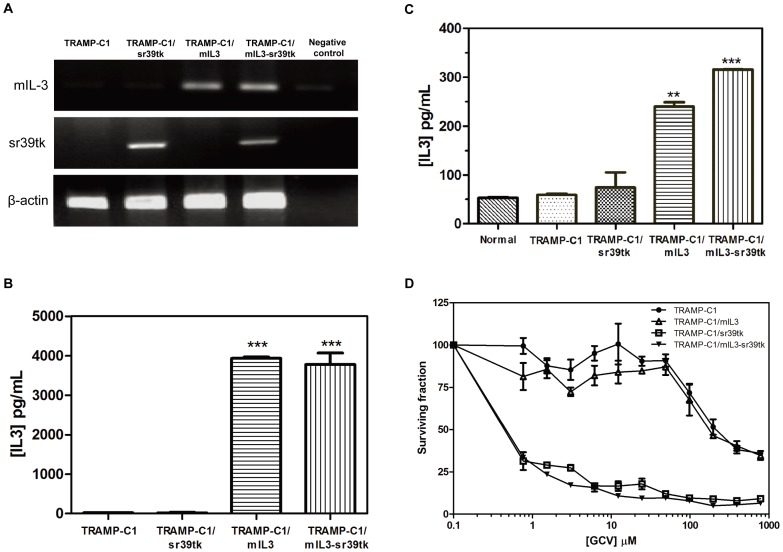
Characterization of HSV-sr39tk and/or mIL-3 gene-expressing murine prostate cancer cell lines. (A) Expression of the HSV-sr39tk and mIL-3 genes *in vitro*. After selection using G-418 for two weeks, gene expression of transfected cell lines was examined by RT-PCR. IL-3 cytokine levels measured in (B) the supernatants of cell lines or (C) blood serum of tumor-bearing mice using ELISA. (D) The cytotoxic effect of the HSV-sr39tk gene transfected into cell lines. Genetically modified cells were seeded in 96 well plates, and then treated with different concentrations of GCV for three days. All data points were analyzed in triplicate and values displayed are means ± SDs. **indicates *P*<0.01, ***indicates *P*<0.001 determined by the Student’s t-test.

To study the effects of combined IL-3 immune therapy and HSV-sr39tk suicide gene therapy, a bicistronic IRES expression vector was used to express both mIL-3 and HSV-sr39tk genes either simultaneously or individually. After selection for two weeks using G-418, gene expression of three transfected cells types, TRAMP-C1/sr39tk, TRAMP-C1/mIL3, and TRAMP-C1/mIL3-sr39tk, was examined by RT-PCR and ELISA. Successful transgenic gene expression was confirmed in all three transfected cell lines as measured by RT-PCR (Fig. 1A). High levels of mIL-3 protein were detected in mIL-3-transfected cell supernatants (Fig. 1B) and in blood serum from mice bearing TRAMP-C1/mIL3 or TRAMP-C1/mIL3-sr39tk tumors (Fig. 1C) as determined using ELISA. To evaluate the sensitivity of transfected cells to the GCV prodrug, cells were incubated in the presence of various concentrations of GCV. TRAMP-C1/sr39tk (IC_50_∶ 0.558 µM) and TRAMP-C1/mIL3-sr39tk (IC_50_∶ 0.575 µM) cells were more sensitive to the cytotoxic action of the GCV prodrug, compared to parental TRAMP-C1 (IC_50_∶ 218.358 µM) and TRAMP-C1/mIL3 cells (IC_50_∶ 179.4 µM) (Fig. 1D). This indicates that the sr39tk protein was expressed in both TRAMP-C1/sr39tk and TRAMP-C1/mIL3-sr39tk cells and the response to GCV was not modified by co-expression of the mIL-3 gene.

### The Anti-tumor Effect of the HSV-sr39tk/GCV System and Co-expressed mIL-3 Gene in a Murine Prostate Cancer Model

**Figure 2:**
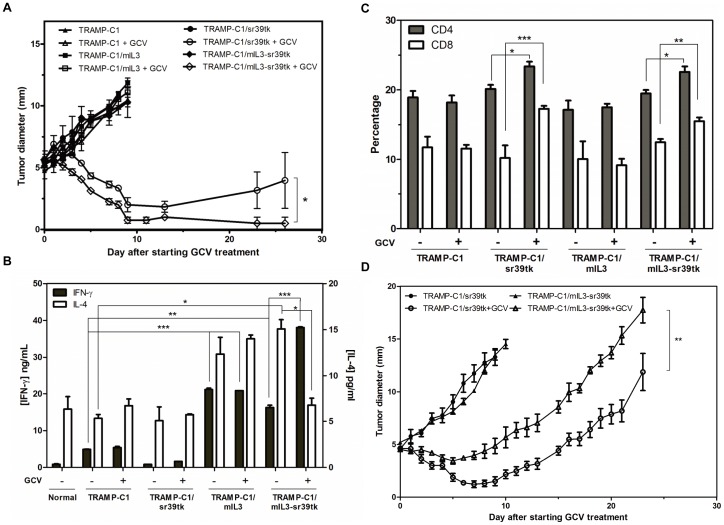
Co-expression of mIL-3 enhanced the anti-tumor effect of the HSV-sr39tk/GCV system and generated a Th1 immune response in an *in vivo* C57BL/6 mouse model of prostate cancer using TRAMP-C1 cells. (A) The response of various gene-transfected TRAMP-C1 prostate tumors to ganciclovir (GCV) treatment. Mice were injected subcutaneously with TRAMP-C1 cells, TRAMP-C1/sr39tk, TRAMP-C1/mIL3 or TRAMP-C1/mIL3-sr39tk transfected cells. When the tumor diameter reached 5 mm, mice were injected intraperitoneally (i.p.) with 33 mg/kg of GCV twice per day for five consecutive days. Tumor sizes were measured every two days. Data points and error bars are means ± SDs for at least three mice per group. (B) Immune responses in the spleens of mice with or without GCV administration as measured by ELISA. (C) Percentages of CD4^+^ and CD8^+^ cells in the spleen after GCV administration as analyzed by flow cytometry. (D) The response of sr39tk-expressing TRAMP-C1 prostate tumors to GCV treatment on SCID mice. Mice were injected subcutaneously with TRAMP-C1/sr39tk and TRAMP-C1/mIL3-sr39tk transfected cells. Therapeutic schedule were as same as the previous experiment. Data points and error bars are means ± SDs for at least three mice per group. *indicates *P*<0.05, **indicates *P*<0.01, ***indicates *P*<0.001 determined by the Student’s t-test.

To determine the anti-tumor effect of combined HSV-sr39tk/GCV and IL-3 therapy, transfected cells were inoculated subcutaneously (s.c.) into the right flanks of mice. Since IL-3-transfected cells do not readily form tumor *in vivo* [20], [22], twice the number of IL-3 expressing cells (TRAMP-C1/mIL3 and TRAMP-C1/mIL3-sr39tk) were used compared to non-IL-3 expressing cells (TRAMP-C1 and TRAMP-C1/sr39tk) for all following *in vivo* experiments. After tumor reached 5 mm in diameter, mice were injected intraperitoneally (i.p.) with GCV. The growth of subcutaneous tumors was markedly suppressed in the TRAMP-C1/sr39tk and TRAMP-C1/mIL3-sr39tk groups following GCV treatment (Fig. 2A). In addition, co-expression of IL-3 was shown to enhance the therapeutic efficacy of the HSV-sr39tk/GCV system. Forty percent of mice tumors regressed completely following combined therapy, while GCV treatment has no effect on the growth rate of the TRAMP-C1 or TRAMP-C1/mIL3 tumors. Three of the mice that had tumor cured were re-challenged by s.c. inoculation of 1×10^6^ viable TRAMP-C1 cells and all resisted the re-challenge (data not shown), indicating that long-term immunity had developed.

To further investigate the immune response initiated following therapy, IFN-γ and IL-4 production by lymphocytes from tumor-bearing mice was measured by ELISA following challenge with irradiated TRAMP-C1 *in vitro*. Lymphocytes isolated from mice bearing TRAMP-C1 or TRAMP-C1/sr39tk tumors did not develop a specific immune response against challenge with irradiated-TRAMP-C1 cells. In contrast, lymphocytes from mice bearing mIL-3-transfected tumors (both TRAMP-C1/mIL3 and TRAMP-C1/mIL3-sr39tk) developed a specific immune response to TRAMP-C1 cells by producing higher levels of IFN-γ and IL-4 (Fig. 2B). In terms of IFN-γ and IL-4 production, the administration of GCV did not alter the immune response of lymphocytes from mice bearing TRAMP-C1, TRAMP-C1/mIL3, or TRAMP-C1/sr39tk tumors. However, GCV administration significantly enhanced IFN-γ, but reduced IL-4 production in lymphocytes from mice bearing TRAMP-C1/mIL3-sr39tk tumors. This indicated that the cytotoxic effects of GCV in sr39tk-transfected cells changed the IL-3-induced immune response towards a Th_1_ response.

Populations of CD4^+^, CD8^+^, and CD4^+^CD25^+^ cells in the spleen were also examined following therapy. Neither HSV-sr39tk nor IL-3 expression influenced the percentage of CD4^+^ or CD8^+^ T cells in splenocytes (Fig. 2C); however, the percentage of CD4^+^ and CD8^+^ T cells in splenocytes of TRAMP-C1/sr39tk- and TRAMP-C1/mIL3-sr39tk-bearing groups was significantly increased following GCV treatment. In contrast, the CD4^+^CD25^+^ cell population in splenocytes was not altered after HSV-sr39tk/GCV therapy or IL-3 expression (Figure S1). These results indicate that HSV-sr39tk/GCV therapy can increase the number of CD4^+^ and CD8^+^ cells in the spleen, but only HSV-sr39tk/GCV therapy combined with mIL-3 gene therapy could lead to development of anti-tumor immunity.

To further confirm the role of T cells in combined therapy, TRAMP-C1/sr39tk and TRAMP-C1/mIL3-sr39tk tumor were subcutaneously implanted into T cell-deficient SCID mice. Without GCV treatment, both tumors grew in the similar rate (Fig. 2D) as they did in normal C57BL/6J mice (Fig. 2A). When tumor grew to 5 mm, mice started to receive GCV treatment. During the period of GCV treatment, tumor size decreased in both single and combined therapies, but they re-grew after GCV treatment was stopped (Figure 2D). This is different from the continue regress of tumors receiving combined treatment in normal C57BL/6J mice (Fig. 2A). This indicated that T cells have critical role on the tumor cure following HSV-sr39tk/GCV single therapy or combined with IL-3 therapy, but are not essential for IL-3 therapy alone. However, we were surprised to find that TRAMP-C1/mIL-3-sr39tk re-grew earlier than TRAMP-C1/sr39tk tumors (Figure 2D; Table 1).

**Table 1:** Relative ratio of tumor diameters after GCV/carrageenan administration or L-NAME administration.

	C57BL/6J			SCID
Group	Control	Carrageenan	L-NAME	T cell-deficient
**TRAMP-C1**	2.8±0.2	2.4±0.1	2.6±0.1	–
**TRAMP-C1/mIL3**	2.6±0.3	2.4±0.1	2.4±0.1	–
**TRAMP-C1/sr39tk**	2.0±0.1	2.3±0.1	2.3±0.1	2.9±0.1
**TRAMP-C1/mIL3-sr39tk**	2.0±0.2	2.2±0.1	2.0±0.3	2.6±0.1
**TRAMP-C1/sr39tk+GCV**	0.9±0.1	1.2±0.2	1.0±0.0	2.6±0.4#
**TRAMP-C1/mIL3-sr39tk+GCV**	0.2±0.1*	2.0±0.2#	0.6±0.1#	3.9±0.3#, *

The diameter of tumors without GCV administration was compared at day nine with the initial diameter (5 mm) at day 0. The diameter of tumors from mice received GCV treatment was compared at day 23 with the initial diameter (5 mm) at day 0 before GCV treatment.

* indicates *P*<0.05 when the TRAMP-C1/sr39tk and TRAMP-C1/mIL3-sr39tk groups were compared.

# indicates *P*<0.05 when the carrageenan, L-NAME and SCID groups were compared with control group. *P* was determined by one way ANOVA test.

### The Role of Macrophages in IL-3-enhanced HSV-tk/GCV Therapy

**Figure 3:**
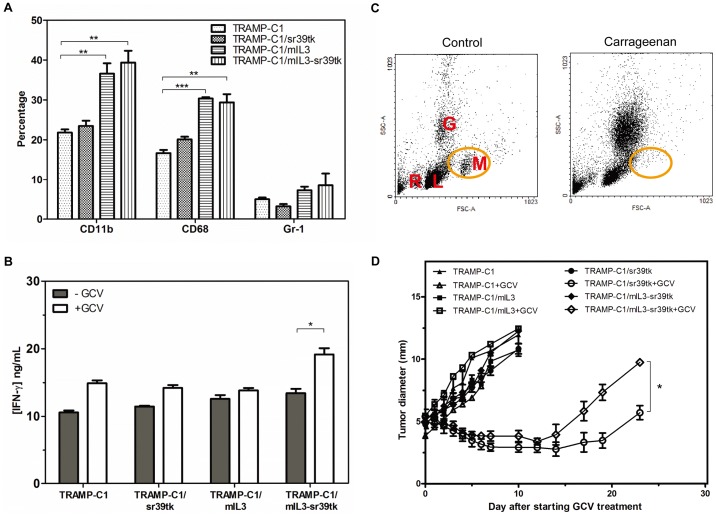
The role of macrophages in IL-3-enhanced HSV-tk/GCV therapy. (A) The percentage of CD11b^+^, CD68^+^, and Gr-1^+^ cells in various gene-transfected tumors was determined by flow cytometry. When a diameter of 6 mm was reached, the tumors were digested to produce a single cell suspension. Tumor-infiltrating cells were studied with flow cytometry. Data and error bars are means ± SDs for n = 3 mice per group. (B) The production of IFN-γ by 2×10^5^ peritoneal macrophages after co-culture with 2×10^5^ transfected cells and treatment with ganciclovir (GCV) for three days. The concentration of IFN-γ in the media was measured by ELISA. Data and error bars are means ± SDs of three independent experiments. (C) Representative flow cytometry profiles to show the changes of splenocyte population after administration of carrageenan. Mice were injected i.p. with carrageenan to deplete macrophages at 7, 3, and 1 day prior to sacrifice. R: red blood cells, L: lymphocytes, M: monocytes, G: granulocytes. (D) The *in vivo* tumor growth curves of various gene-transfected TRAMP-C1 tumors following GCV treatment in carrageenan-treated mice. Mice were injected i.p. with carrageenan to deplete macrophages at 7, 3, and 1 day prior to tumor inoculation, and this continued twice per week after tumor implantation until mice were sacrificed. GCV was injected twice per day for five continuous days when tumors reached 5 mm in diameter. Tumor sizes were measured every two days. Data and error bars are means ± SDs for at least three mice per group. *indicates *P*<0.05, **indicates *P*<0.01, ***indicates *P*<0.001 determined by the one way ANOVA.

Several studies have demonstrated that macrophages are the prime cells responsible for the IL-3-associated anti-tumor T cell response [16], [17]. The results above demonstrate that HSV-sr39tk/GCV therapy altered and enhanced the IL-3-mediated immune response. To examine the role of macrophages in these treatments, the population of tumor-associated macrophages (TAMs) within tumors was initially examined by flow cytometry. Results indicated that HSV-sr39tk gene expression did not alter the number of TAMs in the tumor (Fig. 3A). However, the CD11b^+^ and CD68^+^ cell populations were significantly increased in IL-3-expressing tumors (TRAMP-C1/mIL3 and TRAMP-C1/mIL3-sr39tk). These findings are consistent with those from our previous study using a fibrosarcoma tumor model, which demonstrated that IL-3 could act as a macrophage attractant [20].

To further evaluate the role of macrophages in the IL-3-associated immune response, *in vitro* IFN-γ cytokine production by peritoneal macrophages was measured after co-cultured with tumor cell lines with or without GCV treatment for three days. Neither HSV-sr39tk/GCV-treated nor mIL-3-expressing cells could enhance IFN-γ production by macrophages; however, GCV treatment of TRAMP-C1/mIL3-sr39tk cells increased IFN-γ production by peritoneal macrophages (Fig. 3B). This suggested that HSV-sr39tk/GCV therapy could enhance IL-3-induced M1 macrophage activation so as to stimulate greater production of IFN-γ, which might have been responsible for the development of the Th_1_ immune response described above.

The role of macrophage in combined therapy was further examined by treating mice with an i.p. injection of carrageenan twice per week to deplete macrophage lineage. This treatment effectively depleted the macrophage population, but also increased the number of granulocytes (Fig. 3C). Carrageenan treatment did not affect the tumor growth of examined tumor cell lines in the absence of GCV, but reduced the therapeutic effects of HSV-sr39tk/GCV applied both individually and in combination with IL-3 (Fig. 3D; Table 1). Interestingly, macrophage depletion did not significantly modify the action of IL-3; however, similar to the result on T-cell deficient mice (Fig. 2D), macrophage depletion also dramatically reduced the efficacy of the combined therapy and had greater effects on combined therapy than on simple HSV-sr39tk/GCV therapy, as tumors receiving combined HSV-sr39tk/GCV and IL-3 therapy regrew earlier than those receiving HSV-sr39tk/GCV therapy only, in carrageenan-treated mice (Fig. 3D; Table 1). This suggested that macrophages are not un-replaceable in IL-3-only therapy, but are essential in the HSV-sr39tk/GCV and combined therapies.

### Gene Expression of Macrophages in HSV-sr39tk and/or mIL3 Tumors

**Figure 4:**
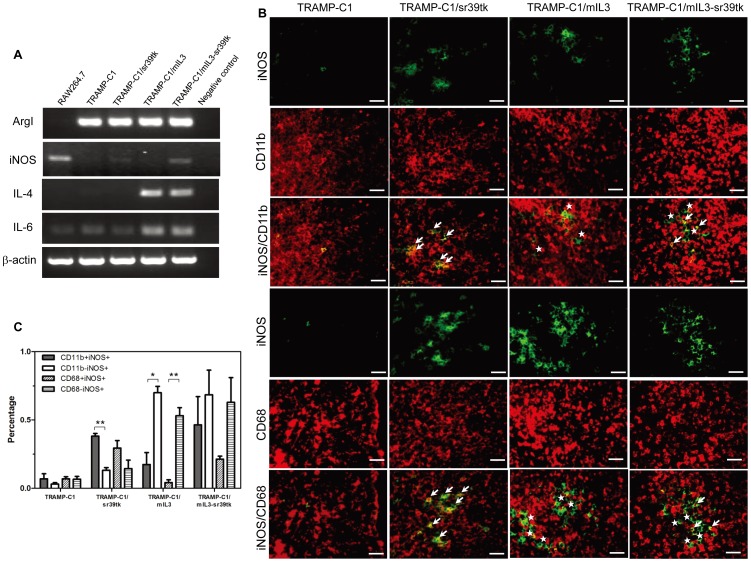
The effect of iNOS inhibition on HSV-sr39tk/GCV and IL-3 therapies. (A) Gene expression of CD11b^+^ cells isolated from tumors as analyzed by RT-PCR. When tumor diameters reached 6–8 mm, tumors were resected and digested to produce a single cell suspension. CD11b^+^ cells were isolated using a MACS CD11b MicroBeads cell separation kit. Total RNA was extracted, reverse transcribed, and amplified with specific primers for 30 cycles. (B) Representative images of iNOS expression from mice in each group. iNOS gene expression was analyzed by immunohistochemical staining. Tumor tissue was stained using anti-iNOS (green) and anti-CD11b/CD68 (red) antibodies. White arrows indicate iNOS^+^/CD11b^+^ or CD68^+^ cells and white stars indicate iNOS^+^/CD11b^−^ or CD68^−^ cells. Original magnification: ×400. Scale bar: 50 µm. Images are representative results from individual animals in each group. (C) Percentages of CD11b^+^iNOS^+^ and CD11b^−^iNOS^+^ were quantified by Image-Pro Plus 6.0 software. *indicates *P*<0.05 determined by the Student’s t-test.

To further explore the role of macrophages in this study, CD11b^+^ cells were isolated from tumors using CD11b antibody-coated magnetic beads and gene expression was analyzed by RT-PCR. All CD11b^+^ cells expressed high levels of ArgI mRNA, indicating that these macrophages were M2-polarized within the tumors. However, CD11b^+^ cells isolated from TRAMP-C1/mIL3 and TRAMP-C1/mIL3-sr39tk tumors showed higher IL-4 and IL-6 gene expression compared to TRAMP-C1 and TRAMP-C1/sr39tk tumors (Fig. 4A). Furthermore, iNOS gene expression by CD11b^+^ cells was found in TRAMP-C1/sr39tk and TRAMP-C1/mIL3-sr39tk tumors, which was verified by immunohistochemical (IHC) staining. The majority of iNOS-positive cells in TRAMP-C1/sr39tk tumors co-expressed CD11b and CD68 (Fig. 4B and C), indicating these cells were TAMs. However, iNOS-positive cells in TRAMP-C1/mIL3-sr39tk tumors were not only found in CD11b^+^ or CD68^+^ TAM cells, but also in CD11b^−^ or CD68^−^ cells. Interestingly, iNOS-positive cells were also found in TRAMP-C1/mIL3 tumors, and the majority did not co-express CD11b or CD68, indicating they were non-TAM cells.

### The Effect of iNOS Inhibition on HSV-sr39tk/GCV and IL-3 Therapies

**Figure 5:**
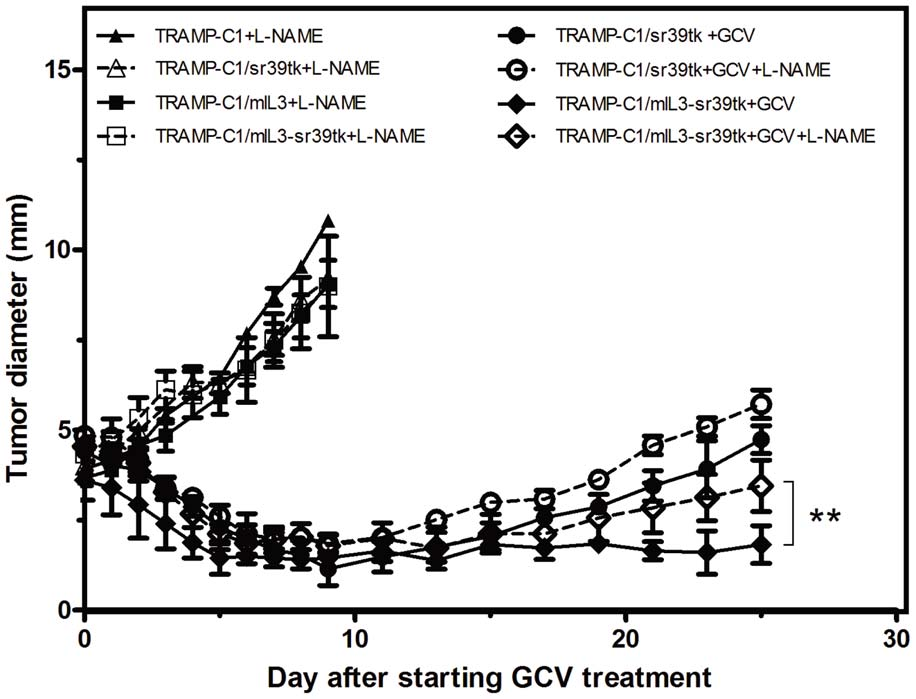
The *in vivo* responses of various gene-transfected TRAMP-C1 tumors in L-NAME-treated mice to GCV treatment. When tumor diameters reached 5 mm, mice were injected i.p. with GCV for five consecutive days and received L-NAME in drinking water for the remaining experimental period. Tumor volumes were measured every two days. Data and error bars are means ± SDs for at least three mice per group. **indicates *P*<0.01 determined by the Student’s t-test.

Interestingly, we observed that HSV-sr39tk and IL-3 gene expression could elevate iNOS expression in TAMs and non-TAMs, respectively (Fig. 4). To explore whether NO production affected the efficacy of HSV-sr39tk/GCV and IL-3 therapies, the iNOS inhibitor, L-NAME, was used to block NO production. When tumors reached 5 mm in diameter, mice were injected i.p. with GCV and received L-NAME via drinking water. Results showed that administration of L-NAME could significantly reduce the efficacy of GCV therapy in TRAMP-C1/mIL3-sr39tk tumors, but not of HSV-sr39tk/GCV or IL-3 therapies individually (Fig. 5; Table 1). This indicated that NO production is not a critical factor for the efficacy of HSV-sr39tk/GCV or IL-3 therapies, but is likely to mediate IL-3-enhancment of HSV-sr39tk/GCV therapy.

## Discussion

In recent years, the suicide gene system has become a well-developed tool for cancer gene therapy. However, its efficacy is still lacking and is generally insufficient to eradicate malignant tumors completely. A number of therapeutic approaches have been proposed to enhance the anti-tumor effect of suicide gene therapy by inducing an immune response to tumor cells via co-expression of cytokine genes, such as IL-2 [23], IL-2 and TNF-α [24], IL-12 [13], and IL-21 [25]. In this study, we demonstrate that bicistronic expression of IL-3 and HSV-sr39tk/GCV suicide gene system exerts a synergistic effect inhibiting the growth of TRAMP-C1 prostate cancer cells *in vivo*, but not *in vitro*. In forty percent of mice tumors were cured by the combined therapy and the cured mice were resisted to the re-challenge with parental tumor, indicating the development of long-term immunity following therapy. Several studies have shown that macrophages are essential cells for the development of IL-3-associated long-term immunity [16], [17], [19], [20], [22], [26]. This study not only provides further support that IL-3 expression can enhance macrophage infiltration, but also demonstrates that macrophages play a critical role in IL-3-enhanced HSV-sr39tk/GCV therapy, as evidenced by reducing the anti-tumor effect of combined therapies in carrageenan-treated mice. Pulaski and colleagues have shown that IL-3 enhances tumor rejection via development of cytotoxic effectors by a mechanism that requires CD4^+^ cells [19], and they demonstrated that IL-3 induced the antigen cross presentation ability of macrophages [27], [28]. In this study, we found that macrophages within IL-3-expressing tumors expressed Arg-1 and increased levels of IL-4 and IL-6, indicating they were M2 TAMs. Interestingly, macrophage depletion by the administration of carrageenan had no effect on IL-3 gene therapy, but reduced the effect of IL-3-enhanced HSV-sr39tk/GCV therapy. The former observation indicates that macrophages may not be the only cells responsible for the effect of IL-3, due to the increase in granulocytes observed in carrageenan-treated mice. However, the latter observation suggests that macrophages are essential for the interaction between HSV-sr39tk/GCV cytotoxic therapy and IL-3, as well as the development of long-term immunity.

The immune response assays demonstrated that expression of the IL-3 gene, alone or co-expression with HSV-sr39tk gene, but not expression of HSV-sr39tk gene alone, can lead to development of specific immune reactions. The splenocytes from mice bearing IL-3-expressing tumors (TRAMP-C1/mIL3 and TRAMP-C1/mIL3-sr39tk) produced both IL-4 and IFN-γ in response to challenge with irradiated-TRAMP-C1 cells. When mice bearing TRAMP-C1/mIL3-sr39tk tumors were treated with GCV, increased IFN-γ production and decreased IL-4 production were found in the splenocytes in response to challenge with irradiated-TRAMP-C1. This may be associated with the development of long-term immunity in these mice and indicates that the function of macrophages within TRAMP-C1/mIL3-sr39tk tumors was altered following GCV administration. The interaction between IL-3-activated macrophages and HSV-sr39tk/GCV cytotoxicity was further illustrated by co-culture assay *in vitro*, which showed enhanced IFN-γ production by peritoneal macrophages in response to GCV in TRAMP-C1/mIL3-sr39tk cells. This suggested that the synergistic interaction between IL-3-activated macrophages and GCV-mediated anti-HSV-sr39tk cell cytotoxicity promoted the development of a Th1 immune response and subsequently contributed to the enhanced anti-tumor activity observed on combining IL-3 and HSV-sr39tk/GCV therapies. The involvement of T cells in HSV-sr39tk/GCV combined IL-3 therapy was further demonstrated by the result that the anti-tumor effect was reduced in SCID mice.

Several studies have demonstrated the occurrence of anti-tumor immunity when tumors were reduced or ablated by HSV-tk/GCV suicide gene therapy. Vile and colleagues reported that the induction of a pronounced intratumoral infiltration by macrophages, CD4^+^ and CD8^+^ T cells was observed after HSV-tk/GCV therapy [29], [30]. In addition, the tumor was converted into an immunostimulatory environment characterized by a Th1 profile in which several cytokines were upregulated, including IL-2, IL-1, IFN-γ and TNF-α. These factors altered the tumor microenvironment leading to necrosis and inflammation, upregulation of costimulatory and adhesion molecules, and induction of an anti-tumor immune response [31], [32]. In this study, we also observed that HSV-sr39tk/GCV therapy could induce massive necrosis (data not shown). Moreover, enhanced proliferation of CD4^+^ and CD8^+^ T cells was observed in the spleen following HSV-sr39tk/GCV therapy. However, unlike that found in other tumor models [29], [30], HSV-sr39tk/GCV therapy alone did not result in development of a Th_1_ immune response or long-term immunity in our TRAMP-C1 prostate tumor model despite that GCV-treated mice having TRAMP-C1/sr39tk re-grew earlier in T cell deficient SCID mice than in immune competent C57BL/6J mice. This discrepancy may be the result of a lack of suitable stimulating factors in the TRAMP-C1 tumor microenvironment, which was overcome when IL-3 was co-expressed.

Our early studies using fibrosarcoma tumor models [20], [22] demonstrated that the expression of the IL-3 gene within tumors could enhance the immunogenicity of classically non-immunogenic tumors, leading to the development of long-term immunity even after the primary tumor was regressed by irradiation [20]. A previous study using a TRAMP-C1 tumor model further demonstrated that irradiation could change the IL-3-driven Th_2_ immune response to a Th_1_ immune response [22]. In the current study, we show additionally that HSV-sr39tk/GCV therapy can convert the IL-3-driven Th_2_ immune response to a Th_1_ immune response. The unique ability of IL-3 to convert the Th_2_-dominant microenvironment into a Th_1_-dominant microenvironment when combined with cytotoxic therapy makes it an ideal immune adjuvant cytokine to cytotoxic therapies such as radiation therapy or chemotherapy. However, it should be noted that it is difficult to form tumors using IL-3-expressing cells alone *in vivo* [19], [20], [27], [28]. In order to form tumors, IL-3-generated immunity was compromised by the inoculation of a larger number of IL-3 expressing tumor cells except the experiment in SCID mice. Therefore, the systemic immune response was suppressed by the overwhelming number of cancer cells even though macrophages were activated by IL-3 [19], [27], [28]. Both radiation and HSV-sr39tk/GCV therapies have the potential to induce extensive tumor destruction. These therapies not only cause tumor regression, but also alleviate immunosuppression and release tumor-associated antigens taken up by IL-3-activated macrophages, which contribute to the enhanced anti-tumor immune response. However, we found that GCV-treated mice carrying TRAMP-C1/mIL3-sr39tk tumors re-grew earlier than TRAMP-C1/sr39tk tumors in either carrageenan-treated macrophage depletion mice or T cell deficient SCID mice. It is likely IL-3 might have tumor promotion role when macrophages or T cells were absent in tumor microenvironment. One may argue why these two tumors had same growth rate in mice without GCV treatment. It may be a matter of cell numbers. The large number of tumor cell inoculation at the beginning may conceal the tumor promotion effect.

Another interesting finding of this study was that NO appeared to play different roles in IL-3 immune therapy and HSV-sr39tk/GCV cytotoxicity. The critical role of NO in tumor progression has been well studied. Within the tumor microenvironment, NO can be generated by macrophages, neutrophils, endothelial cells, fibroblasts, and, in certain cases, by tumor cells themselves. NO has been hypothesized to play a dual role within the tumor microenvironment. Despite its established role in anti-tumor responses [33], [34], in certain circumstances, NO production promotes tumor progression [35]. The local concentration of NO may partially explain its biphasic nature in cancer. In this study, we found that NO production by cells varied depending on tumor type. iNOS-positive cells were rarely detected in control tumors. In tumors carrying the HSV-sr39tk gene (TRAMP-C1/sr39tk tumor), the iNOS-positive cells were mainly CD11b^+^ TAMs, but in tumors carrying IL-3 gene (TRAMP-C1/mIL3 tumor), the iNOS-positive cells were mainly CD11b^−^ non-TAMs. Since TRAMP-C1/mIL3-sr39tk tumors express both IL-3 and HSV-sr39tk genes, iNOS-positive cells were found in both TAMs and non-TAM cells. It is unclear how iNOS were induced in these gene-transfected tumors. Nevertheless, we observed that administration of the iNOS inhibitor, L-NAME, diminished the combined therapeutic effects of HSV-sr39tk/GCV and IL-3, but had no effect on HSV-sr39tk/GCV prodrug therapy or IL-3 immune therapy individually. This suggests that NO may play an important role in the interaction between the cytotoxic effect of the HSV-sr39tk/GCV system and the IL-3-mediated immune reaction. However, it may be just an issue of the level of NO production, since TRAMP-C1/mIL3-sr39tk tumors produced more NO than TRAMP-C1/sr39tk or TRAMP-C1/mIL-3 tumors.

In summary, we have demonstrated that co-expression of IL-3 can enhance the cytotoxic effects of HSV-sr39tk/GCV prodrug therapy, and this could convert the IL-3-directed Th_2_ response into a Th_1_ immune response. Furthermore, we showed that the interaction between IL-3-activated macrophages and HSV-sr39tk/GCV therapy is associated with NO production by TAMs. The current study suggests a feasible strategy by which immune gene therapy in conjunction with a suicide gene system can significantly improve the efficacy of prostate cancer treatment. Importantly, prodrug-based suicide gene therapy possesses the added benefits of reduced side effects on normal tissue and increased specificity for tumor cells.

## Supporting Information

Figure S1 **Percentage of CD4^+^CD25^+^ cells in the spleen after GCV administration as determined by flow cytometry.**
(TIF)
