# Supplementary figures and images for: The Roles of Macrophages and Nitric Oxide in Interleukin-3-Enhanced HSV-Sr39tk-Mediated Prodrug Therapy
Source: PLoS One. 2013 Feb 18;8(2):e56508. doi: 10.1371/journal.pone.0056508 (PMC3575414; doi:10.1371/journal.pone.0056508)

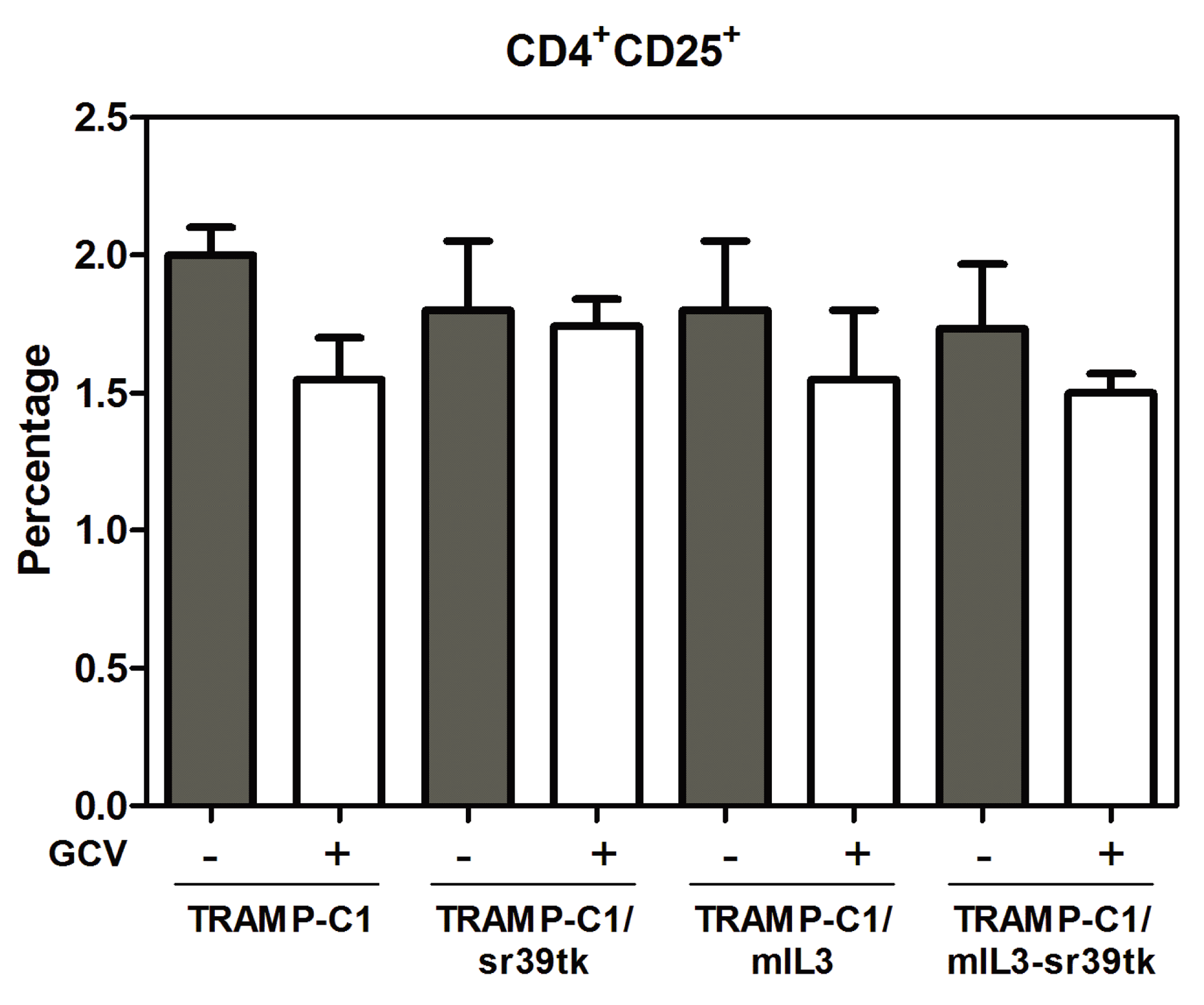

Supplement: Figure S1 — Percentage of CD4+CD25+ cells in the spleen after GCV administration as determined by flow cytometry. (TIF) [file pone.0056508.s001.tif]
